# Interferon-γ secreted by recruited Th1 cells in peritoneal cavity inhibits the formation of malignant ascites

**DOI:** 10.1038/s41420-023-01312-5

**Published:** 2023-01-23

**Authors:** Chang Liu, Zhuanglong Xiao, Li Du, Shenghua Zhu, Hongyu Xiang, Zehui Wang, Fang Liu, Yuhu Song

**Affiliations:** 1 Division of Gastroenterology, Union Hospital, Tongji Medical College, Huazhong University of Science and Technology, Wuhan, 430022 China; 2 Institute of Hematology, Union Hospital, Tongji Medical College, Huazhong University of Science and Technology, Wuhan, 430022 China

**Keywords:** Cancer immunotherapy, Cancer microenvironment

## Abstract

Type 1 T helper (Th1) cells generate an efficient antitumor immune response in multiple malignancies. The functions of Th1 cells in malignant ascites (MA) have not been elucidated. The distribution of helper T cells in peritoneal fluid and peripheral blood was determined in patients and animal models with malignant ascites. The effects of Th1-derived interferon-γ (IFN-γ) on the formation of malignant ascites were investigated. The mechanism underlying the recruitment of Th1 cells into peritoneal cavity was explored. In patients with malignant ascites and animal models of malignant ascites, the percentage of Th1 cells increased in peritoneal fluid compared with peripheral blood. Next, our experiment demonstrated that Th1 cells inhibited the growth of tumor cells by secreting IFN-γ in vitro. In murine models of malignant ascites, increased peritoneal fluid and shorter survival time were observed in IFN-γ^−/−^ mice compared with wild-type (WT) mice. Then, the levels of C-X-C motif chemokine ligand (CXCL) 9/10 and the ratio of CXCR3^+^ Th1 cells indicated the involvement of CXCL9, 10/CXCR3 axis in the recruitment of Th1 cells into peritoneal cavity. As expected, in murine models of malignant ascites, the gradient between ascitic Th1 ratio and blood Th1 ratio decreased in CXCR3^−/−^ mice compared with WT mice. IFN-γ secreted by recruited Th1 cells in peritoneal cavity inhibits the formation of malignant ascites. Hence, manipulation of Th1 cells or IFN-γ will provide a therapeutic candidate against malignant ascites.

## Background

Malignant ascites account for about 5–25% of all cases of ascites and carry a poor prognosis. Malignant ascites is commonly associated with a variety of neoplasms containing gastric, colorectal, pancreatic, hepatobiliary, ovarian, endometrial, primary peritoneal carcinomas [1–5]. Obviously, the formation of malignant ascites is a complex, multifactorial process. An imbalance between fluid secretion and absorption by peritoneum contributes to abnormal accumulation of fluid within peritoneal cavity. Previous studies revealed malignant ascites results from the alteration in vascular permeability, the release of inflammatory cytokines and the obstruction of lymphatic drainage [6, 7]. However, the pathophysiology of malignant ascites has been incompletely understood. Thus, further researches should be performed to explore the mechanism.

The immune system can distinguish between normal cells and abnormal cells, and plays a critical role in fighting cancer. T lymphocytes, an essential part of immune system, are found in and around tumors, and seem to be critical in determining the efficacy of immune surveillance [8, 9]. Helper T cells play a central role in normal immune response by releasing factors that activate virtually all the other immune system cells [10, 11]. Helper T cells are arguably important cells in adaptive immunity. Recent studies demonstrated help T cell serves as a key mediator through inducing efficient antitumor immune response [10, 12]. Helper T cells differentiate into Type 1 helper T (Th1), Th2, Th17, and regulatory T (Treg) cells by exposure to various cytokines and cellular interactions [11, 12]. Th1 cell has been considered as an efficient CD4^+^ T cell subset to generate antitumor immune response through different ways [9, 12]. It has been proven that Th1 cells participated in the formation of malignant pleural effusion [13–15]. Thus, we hypothesized Th1 cells participate in the pathogenesis of malignant ascites. However, the role of Th1 in malignant ascites was not reported yet. The function of Th1 cells in the formation of malignant ascites were determined, and the mechanism underlying the recruitment of Th1 cells into peritoneal cavity was explored in our study. Our results demonstrated interferon γ produced by Th1 cell participated in the formation of malignant ascites through suppressing the growth of the tumor and reducing peritoneal permeability in vivo, and CXCL9,10/CXCR3 axis mediated the recruitment of Th1 cells into peritoneal cavity through visceral peritoneum.

## Results

### Significant increase of Th1 cells in malignant ascites

**Fig. 1:**
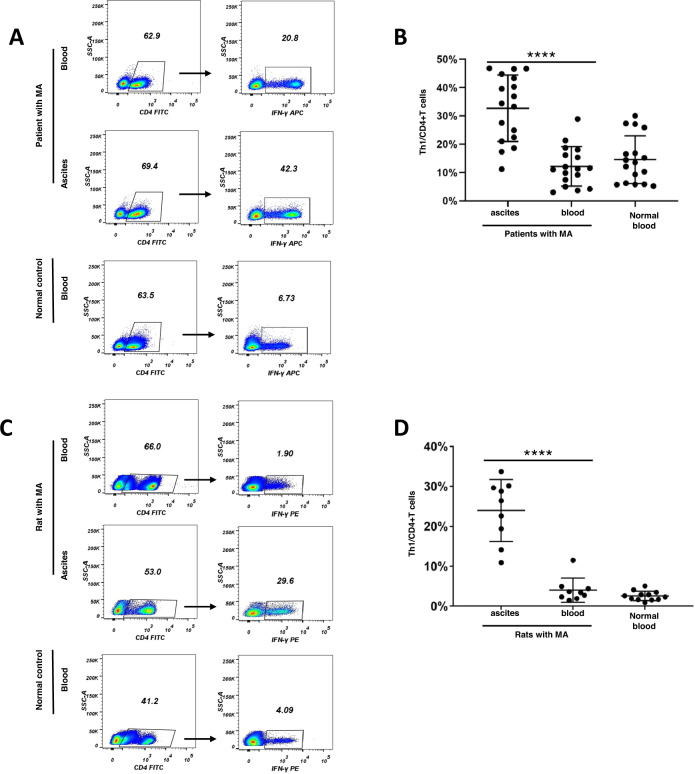
Th1 cells in human and rat malignant ascites. **A** Representative dot plots from a patient with malignant ascites (MA) and a healthy control showing flow cytometric analysis of Th1 (IFN-γ^+^CD4^+^) cells; **B** the percentages of Th1 cells in peritoneal fluid, peripheral blood in patients with malignant ascites. The difference between two groups was determined by *t* test; **C** representative flow cytometric dot plots of Th1 cells from a rat with malignant ascites at 21 days after intraperitoneal injection of Walker-256 cells; **D** the percentages of Th1 cells in peritoneal fluid, peripheral blood in rat model of malignant ascites. The difference between two groups was analyzed by *t* test. ****p ≤ 0.0001.

Growing evidence has demonstrated helper T cells play important roles in tumor immune surveillance [9, 12, 16]. Thus, the distribution of help T cells was initially determined in patients and animal models with malignant ascites. Firstly, different subtypes of help T cells (Th1, Treg, Th2, Th17) in peripheral blood and peritoneal fluid were determined in patients with malignant ascites. Significant increases in Th1 cells (CD4^+^IFN-γ^+^), Treg cells (CD4^+^CD25^+^CD127^−^), and Th17 cells (CD4^+^IL-17^+^) were observed in malignant ascites compared with the corresponding blood (Fig. 1A, B and Fig. S1 and S2). Simultaneously, the results showed no significant differences in the percentage of Th2 cells (CD4^+^ IL-4^+^) between peritoneal fluid and corresponding blood (Fig. S3). Secondly, the accumulation of peritoneal fluid and the tumors in peritoneal cavity were observed in rat model of malignant ascites (Fig. S4). In addition, the level of vascular endothelial growth factor (VEGF) and angiotensin 2 (Ang-2), key cytokines of peritoneal permeability, increased in peritoneal fluid compared with peripheral blood (Fig. S5). All these confirmed that rat model of malignant ascites was established successfully. The results of flow cytometry (Fig. 1C, D) showed significant increase of Th1 cells in peritoneal fluid compared with the corresponding blood in rat model of malignant ascites. Importantly, the proportions of different Th cell subtypes were analyzed in malignant ascites. In patients with malignant ascites, 32.7 % of CD4^+^ lymphocyte was Th1 cell, 11.5% of CD4^+^ lymphocyte was Treg cell, 1.8% of CD4^+^ lymphocyte was Th2 cell; 2.6% of CD4^+^ lymphocyte was Th17 cell (Fig. 1, Figs. S1–3). Interestingly, similar ratio of Th1 cells in peritoneal fluid was observed in rat model of malignant ascites (Fig. 1C, D). These indicated Th1 cells accounted for the largest subtype of helper T cells in malignant ascites. In addition, the gradient between ascitic Th1 ratio and blood Th1 ratio was large. Given these, Th1 cells were selected as the target subtype of helper T cells in the pathogenesis of malignant ascites in our study.

### Th1 cells inhibits the growth of tumor cells used for murine model of malignant ascites by secreting IFN-γ in vitro

**Fig. 2:**
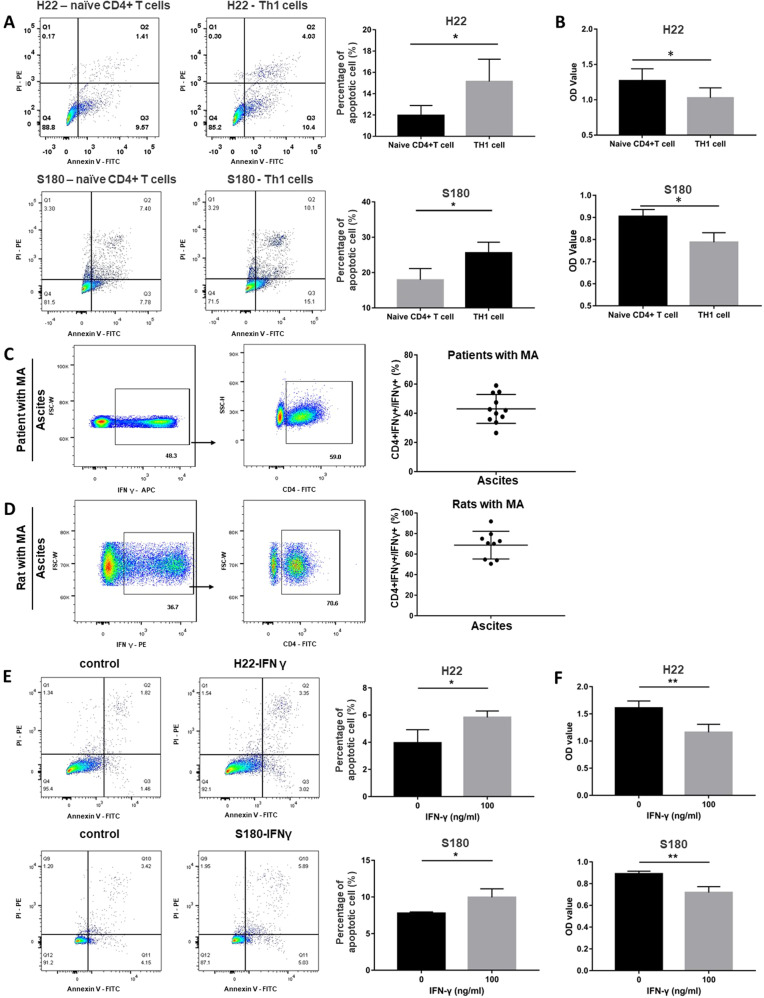
Th1 cells inhibit the growth of tumor cells for malignant ascites by secreting IFN-γ. **A** Flow cytometric analysis revealed that Th1 cells induced apoptosis of H22 cells and S180 cells in vitro after 48-h incubation with Th1 cells. Upper panel: representative flow cytometric dot plots of apoptosis assay (left) and the percentages of apoptotic cells (right) revealing Th1 cells induced apoptosis of H22 cells in vitro; lower panel: representative flow cytometric dot plots of apoptosis assay (left) and the percentages of apoptotic cells (right) revealing Th1 cells induced apoptosis of S180 cells in vitro. The difference between two groups was determined by *t* test. **B** proliferative activity assay showing Th1 cells suppressed the proliferation of H22 cells (upper) and S180 cells (lower). The difference between two groups was determined by *t* test. **C** Representative dot plots of flow cytometric analysis showing the percentage of CD4^+^ cells in IFN-γ-producing cells in a patient with malignant ascites (left); the percentage of CD4^+^ cells in IFN-γ-producing cells in patients with malignant ascites (right); **D** Representative dot plots of flow cytometric analysis showing the percentage of CD4^+^ cells in IFN-γ-producing cells in a rat with malignant ascites (left); the percentage of CD4^+^ cells in IFN-γ-producing cells in rats with malignant ascites (right). **E** Flow cytometric analysis revealed that IFN-γ induced apoptosis of H22 cells and S180 cells in vitro after 48-h incubation with IFN-γ. Upper panel: representative flow cytometric dot plots of apoptosis (left) and the percentage of apoptotic cells (right) revealing IFN-γ induced the apoptosis of H22 cells in vitro; lower panel: representative flow cytometric dot plots of apoptosis assay (left) and the percentage of apoptotic cells (right) revealing IFN-γ induced the apoptosis of S180 cells in vitro. The difference between two groups was determined by *t* test; **F** proliferative activity assay showing IFN-γ suppressed the proliferation of H22 cells (upper) and S180 cells (lower). The difference between two groups was determined by *t* test. *p ≤ 0.05, **p ≤ 0.01, ***p ≤ 0.001.

Since the mice with a genetic disruption of Th1 cells is not available, the effects of Th1 cells on the growth of tumor cells used for establishing murine model of malignant ascites were determined in vitro. Naive CD4^+^ T cells were purified from mouse spleen instead of malignant ascites since it is difficult to obtain sufficient number of naïve T cells from malignant ascites. Then, naive T cells were differentiated into Th1 cells in the presence of Th1 conditions [13]. Our results showed the purity of naïve CD4^+^ T cells was 92.6%, and the purity of Th1 cells reached 93.4% (Fig.S6). Subsequently, the tumor cells (H22 cells and S180 cells) used for murine models of malignant ascites were co-cultured with naive CD4^+^ T cells or Th1 cells for 48 h; and the apoptosis and proliferative activity of tumor cells were evaluated. Apoptosis assay (Fig. 2A) showed that the proportion of apoptotic cells increased remarkably in tumor cells (H22 cells and S180 cells) treated with Th1 cells compared with naïve CD4^+^ T cells (*p* < 0.05). Proliferation assay (Fig. 2B) revealed that proliferative activity of tumor cells was inhibited upon the treatment of Th1 cells. These results revealed that Th1 cells inhibited the growth of H22 and S180 tumor cells in vitro. Interferon γ is a hallmark of Th1 lymphocytes, and IFN-γ promotes the development and function of Th1 cells [12, 17–19]. Then, the percentage of CD4^+^ cell in IFN-γ-producing cells was determined in ascites. Our results showed 43.0% of IFN-γ-producing cells were Th1 cells in patients with malignant ascites. In rat model of malignant ascites, 68.8% of IFN-γ-positive cells were Th1 cells (Fig. 2C, D). These indicated critical role of Th1 in the production of IFN-γ in malignant ascites. Then, the effects of IFN-γ on the growth of tumor cells used for murine models of malignant ascites were investigated. As shown in Fig. 2E, F, the proportion of apoptotic cells increased (Fig. 2E) and proliferative activity of tumor cells decreased (Fig. 2F) upon the incubation of IFN-γ, indicating the tumor suppressive effect of IFN-γ. These revealed that Th1 cells inhibited the growth of tumor cells by secreting IFN-γ.

### Interferon γ inhibits the formation of malignant ascites in vivo

**Fig. 3:**
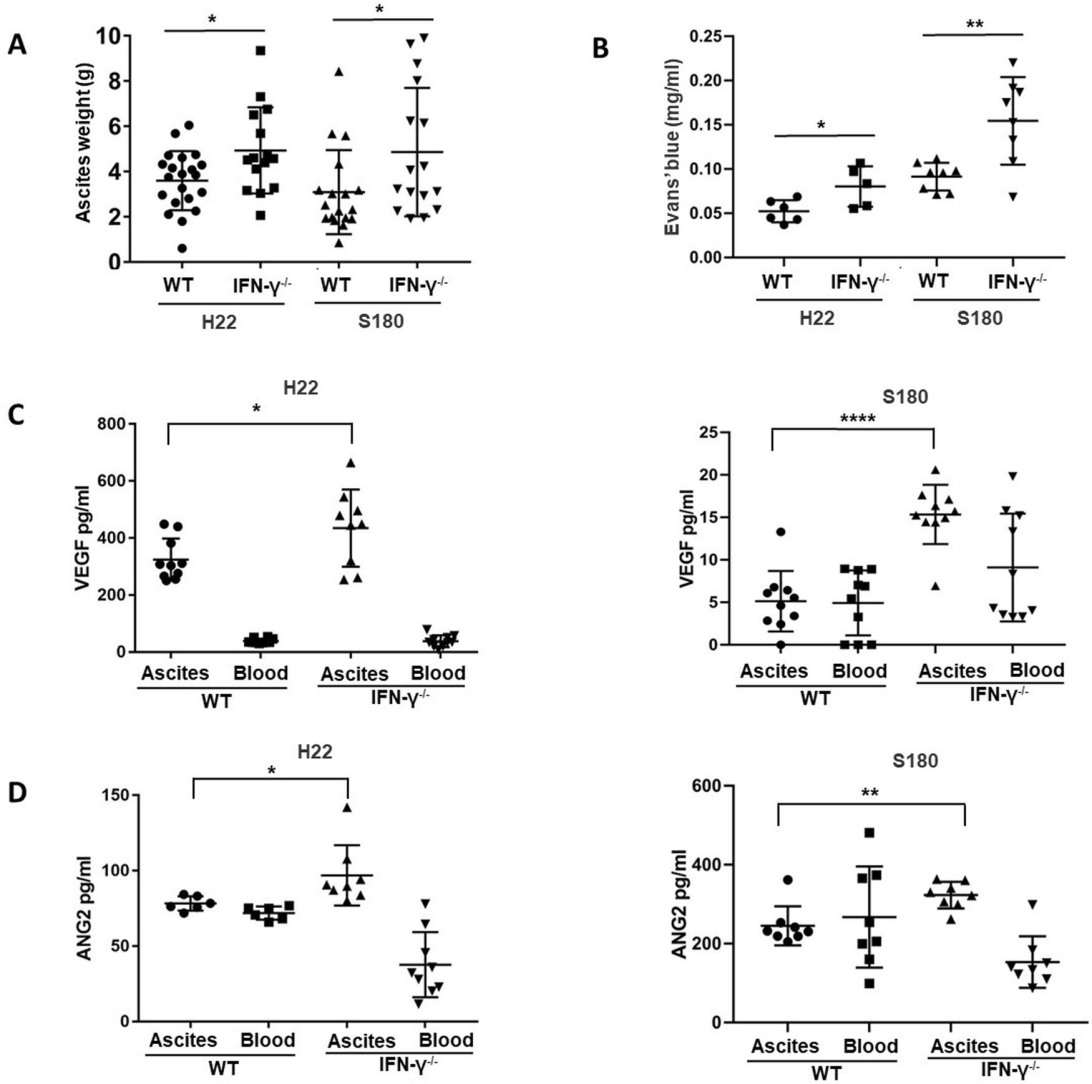
Effects of IFN-γ on the formation of malignant ascites. **A** The weight of peritoneal fluid at 14 days after intraperitoneal injection of H22 or S180 cells showed that deficiency of IFN-γ promoted the formation of peritoneal fluid in murine model of malignant ascites; **B** peritoneal permeability assay demonstrated IFN-γ deficiency increased peritoneal permeability through determining Evan’s blue; **C** the concentration of VEGF in peritoneal fluid and corresponding blood of WT or IFN-γ^−/−^ mice with malignant ascites; left panel: H22; right panel: S180; **D** the concentration of angiotensin-2 in peritoneal fluid and corresponding blood of WT or IFN-γ^−/−^ mice with malignant ascites; left panel: H22; right panel: S180. The difference between two groups was determined by *t* test. *p ≤ 0.05, **p ≤ 0.01, ***p ≤ 0.001, ****p ≤ 0.0001, ns no significant.

**Fig. 4:**
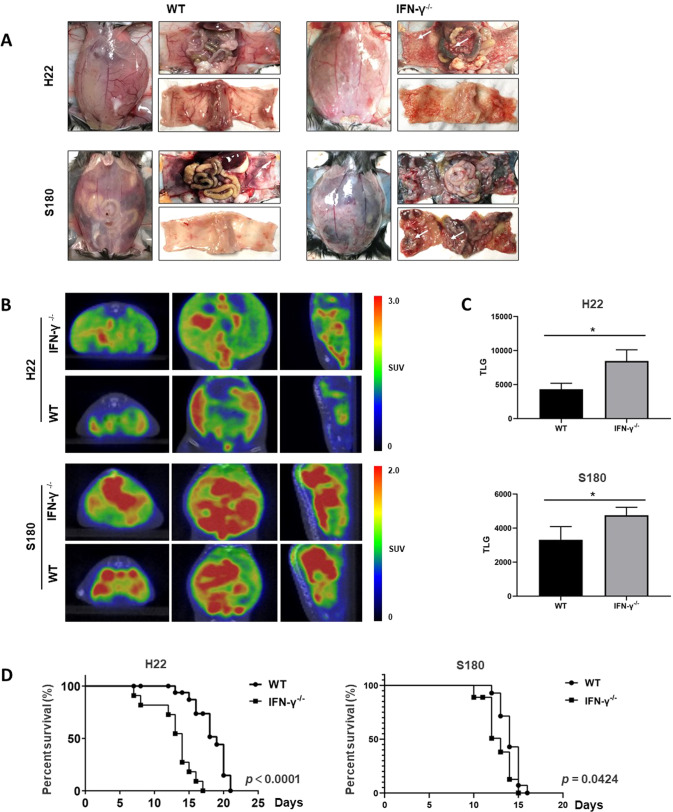
Effects of IFN-γ on the growth of peritoneal carcinomatosis and the survival of mice. **A** Representative photograph of WT and IFN-γ−/− mice after intraperitoneal injection of H22 and S180 cells. Marked abdomen expansion and multiple tumor foci were observed at 14 days after the administration of tumor cells. Representative photography showed most of tumor cells were found in visceral peritoneum; **B** positron emission tomography and computed tomography imaging of WT and IFN-γ^−/−^ mice with malignant ascites; **C** total lesion glycolysis (TLG) on PET/CT of WT and IFN-γ^−/−^ mice with malignant ascites. The difference between two groups was determined by *t* test; **D** survival curve of WT and IFN-γ^−/−^ mice with malignant ascites. The difference between two groups was determined by pairwise log-rank tests; *p ≤ 0.05, **p ≤ 0.01, ***p ≤ 0.001, ns no significant.

The above data have demonstrated Th1 suppressed the growth of tumor cells via IFN-γ in vitro, thus, the effect of IFN-γ on the formation of malignant ascites was determined in vivo. Murine models of malignant ascites were created by injecting of H22/S180 cells into IFN-γ^−/−^ mice or the control. The accumulation of peritoneal fluid and peritoneal carcinomatosis were visible in IFN-γ^−/−^ mice and the control (wild-type) (Figs. 3A, 4A). The weight of peritoneal fluid increased in IFN-γ^−/−^ mice compared with the control mice (Fig. 3A). Then, peritoneal permeability was evaluated since the increase of peritoneal permeability resulted in the formation of ascites. Peritoneal permeability was evaluated by the leakage of Evan’s blue into peritoneal cavity. As shown in Fig. 3B, the concentration of Evan’s blue in ascites increased significantly in IFN-γ^−/−^ mice compared with the control (wild-type). Finally, the levels of VEGF and ANG-2, key regulators of peritoneal permeability, were determined. The results (Fig. 3C, D) showed the concentration of VEGF and ANG-II in peritoneal fluid increased in IFN-γ^−/−^ mice compared with the control. Therefore, murine models of malignant ascites using IFN-γ^−/−^ mice demonstrated that IFN-γ inhibited the formation of malignant ascites.

### IFN-γ suppresses the growth of peritoneal carcinomatosis in vivo

In murine models of malignant ascites, peritoneal tumors were found to reside on peritoneum in WT, IFN-γ^−/−^mice administrated with H22 cells. The numbers of peritoneal tumor foci increased in the IFN-γ^−/−^mice group compared with the control (Fig. 4A, upper panel). To evaluate the presence of peritoneal carcinomatosis in the living mice, PET-CT scan was performed in murine models of malignant ascites. Increased radiotracer uptake on FDG-PET scanning indicated tumors (Fig. 4B, upper panel). The PET imaging confirmed total lesion glycolysis increased in IFN-γ^−/−^mice group compared with the control (Fig. 4C, upper panel). Interestingly, similar patterns were observed in WT, IFN-γ^−/−^mice injected with S180 cells (Fig. 4A–C, bottom panels). In addition, the survival of murine models was evaluated. The median survival times of WT, IFN-γ^−/−^mice bearing malignant ascites induced by the administration of H22 cells were 18 and 14 days, respectively. Pairwise log rank tests showed that there was significant difference in survival curve between IFN-γ^−/−^ group and WT mice administrated with H22 cells (Fig. 4D, left panel). Similar patterns were observed in WT, IFN-γ^−/−^mice injected with S180 cells (Fig. 4D, right panel). All these showed that IFN-γ deficiency promoted the development of peritoneal cancer and decreased the survival of mice with malignant ascites.

### The recruitment of CXCR3^+^ Th1 cells into peritoneal cavity via chemokine CXCL9 and CXCL10

**Fig. 5:**
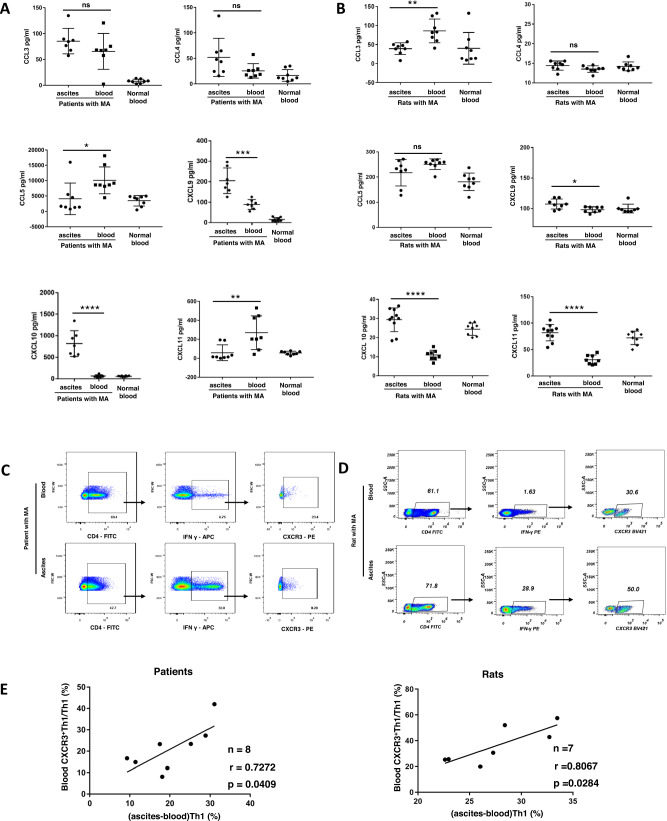
The expression of chemokines and receptors for recruitment of Th1 cells in patients and rats. **A** Th1-chemotaxin-related chemokines (CCL3, CCL4, CCL5, CXCL9, CXCL10, CXCL11) in peritoneal fluid were determined by ELISA in patients with malignant ascites (MA). The difference between two groups was determined by *t* test; **B** the concentrations of Th1-chemotaxin-related chemokines (CCL3, CCL4, CCL5, CXCL9, CXCL10, CXCL11) in peritoneal fluid in rat models of malignant ascites. The difference between two groups was determined by *t* test.; **C** representative flow cytometric dot plots showing CXCR3^+^ Th1 cells in malignant ascites of patients compared with peripheral blood; **D** representative flow cytometric dot plots showing CXCR3^+^ Th1 cells in malignant ascites of rat model compared with peripheral blood; **E** statistical analysis showing positive correlation between the ratio of serum CXCR3^+^ Th1 cells, and ascites-blood gradient of Th1 ratio in patients (left) and rat models (right). The correlations were analyzed by Pearson correlation. *p ≤ 0.05, **p ≤ 0.01, ***p ≤ 0.001, ****p ≤ 0.0001, ns no significant.

**Fig. 6:**
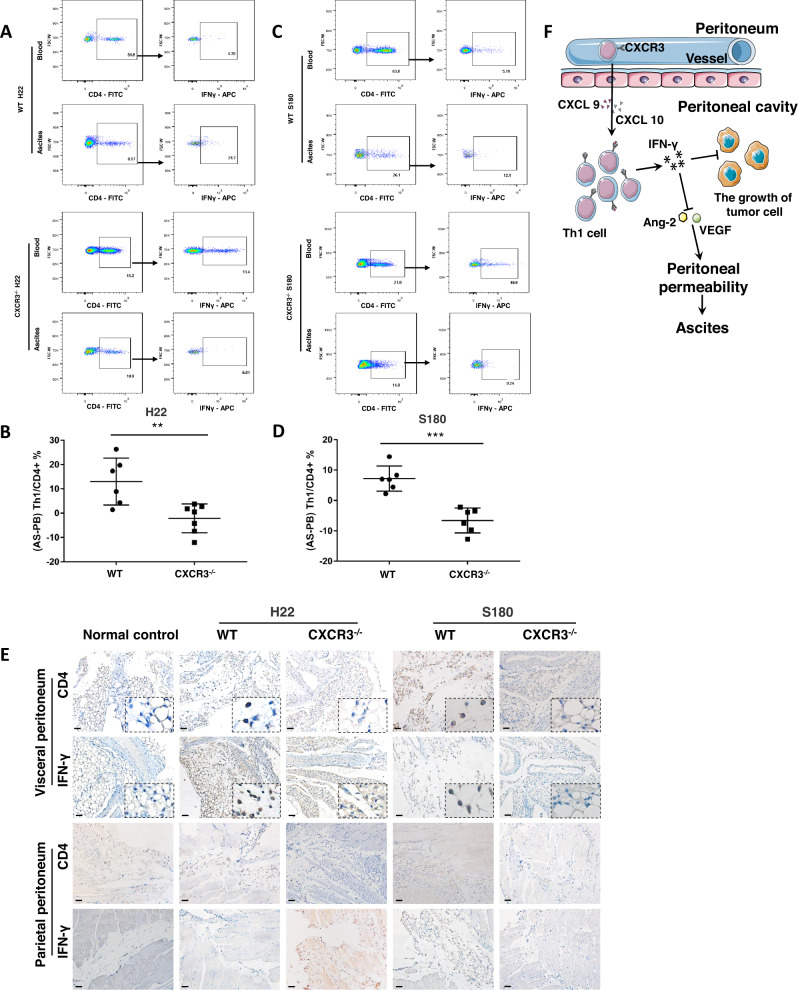
CXCR3 plays a key role in the migration of Th1 cells into peritoneal cavity. **A** the representative flow cytometric dot plots of Th1 cells in peritoneal fluid and peripheral blood from WT and CXCR3^−/−^ mice at 14 days after intraperitoneal injection of H22 cell; **B** the ascites-peripheral blood gradient of Th1 ratio from WT and CXCR3^−/−^ mice at 14 days after intraperitoneal injection of H22 cell. The difference between two groups was determined by *t* test; **C** representative flow cytometric dot plots of Th1 cells in peritoneal fluid and peripheral blood from WT and CXCR3^−/−^ mice at 14 days after intraperitoneal injection of S180 cell; **D** the ascites-peripheral blood gradient of Th1 ratio from WT and CXCR3^−/−^ mice at 14 days after intraperitoneal injection of S180; the difference between two groups was determined by *t* test; **E** immunohistochemical staining revealed that a decrease of Th1 cells (CD4 + IFN-γ+) in visceral peritoneum of CXCR3^−/−^ mice compared with wild-type mice; **F** Graphical summary of present research: CXCL9,10/CXCR3 axis mediated the recruitment of Th1 cells into peritoneal cavity, interferon γ secreted by Th1 cells in peritoneal fluid suppressed the growth of tumor cells. In addition, interferon γ reduced peritoneal permeability via VEGF and Ang-2. *p ≤ 0.05, **p ≤ 0.01, ***p ≤ 0.001, ns no significant; scale bars = 50 μm.

Leukocyte recruitment is regulated by chemokines and their corresponding chemokine receptor expressed in leukocyte [20, 21]. Therefore, we determined chemokines and chemokine receptors involved in the recruitment of Th1 cells into peritoneal cavity. The samples of peripheral blood and ascites were collected from patients, and then Th1-chemotaxin-related chemokines (chemokine C-C motif ligand (CCL)3, CCL4, CCL5, CXCL9, CXCL10, CXCL11) in peripheral blood and peritoneal fluid were determined (Fig. 5A). The results of ELISA showed the concentrations of the chemokines CXCL9 and CXCL10 were higher in malignant ascites than those in peripheral blood (Fig. 5A). Since CXCR3 is the corresponding receptor for CXCL9 and CXCL10, CXCR3 expression was determined in Th1 cells. Interestingly, the correlation analysis showed the ratio of CXCR3^+^ Th1 cells in peripheral blood correlated positively with the gradient between ascitesTh1 ratio and blood Th1 ratio (Fig. 5C, E, left panel). To further confirm our findings, chemokines and chemokine receptors in peripheral blood and ascites were determined in rat model of malignant ascites, similar results were found in rat model of malignant ascites (Figs. 5B, D, E, right panel). The above results indicated that the CXCL9, 10/CXCR3 axis plays a key role in the migration of Th1 cells into peritoneal cavity. To prove the key role of CXCR3, CXCR3 deficient mice received the administration of tumor cells to establish murine models of malignant ascites. As expected, in murine models of malignant ascites, the gradient between ascites Th1 ratio and blood Th1 ratio was lower in CXCR3^−/−^ mice compared with wild-type mice (Fig. 6A–D); which indicated critical role of CXCR3 in the migration of Th1 cells into peritoneal cavity. Another important issue is how Th1 cells in peripheral blood streamed into malignancy-affected peritoneal cavity. In rat model of malignant ascites, immunohistochemical staining of CD4 and IFN-γ showed Th1 cells were preferentially located in visceral peritoneum, not in parietal peritoneum. It indicated Th1 cell recruited into peritoneal cavity through visceral peritoneum (Fig. S7). Then, the results of immunochemical staining revealed that a decrease in the number of Th1 cells was observed in visceral peritoneum of CXCR3^−/−^ mice compared with wild-type mice (Fig. 6E). While, no significant change in the number of Th1 cells was shown in parietal peritoneum. These findings suggest that CXCR3 plays an important role in the recruitment of Th1 cells into peritoneal cavity through visceral peritoneum. However, the increase in the numbers of peritoneal tumor foci and the weight of peritoneal fluid was not found in CXCR3^−/−^ mice compared with wild-type mice (Fig. S8 and S9). All these revealed the recruitment of Th1 cells into peritoneal cavity via CXCL9, 10/CXCR3 axis.

## Discussion

Malignant ascites is a common manifestation in the late stage of gastrointestinal tract cancers and ovarian cancer [5]. Previous studies demonstrated Th1 cells mediated anti-cancer immunity through different ways [9, 22]. While, the role of Th1 cells in malignant ascites has not been explored until now. Thus, the details of Th1 cells in malignant ascites were investigated in our study. The accumulation of Th1 cells in peritoneal fluid was observed in patients with malignant ascites and animal model of malignant ascites, and Th1 cells inhibited the growth of tumor cells by secreting IFN-γ in vitro. Interferon γ participated in the formation of malignant ascites through suppressing the growth of the tumor and reducing peritoneal permeability in vivo. Importantly, further study demonstrated CXCL9, CXCL10/CXCR3 axis played an important role in the recruitment of Th1 cells into peritoneal cavity. In conclusion, our results demonstrated interferon γ produced by Th1 cell participated in the formation of malignant ascites through suppressing the growth of the tumor and reducing peritoneal permeability in vivo, and CXCL9,10/ CXCR3 axis mediated the recruitment of Th1 cells into peritoneal cavity through visceral peritoneum (Fig. 6F).

CD4^+^ T lymphocytes within the tumor microenvironment (TME) play important roles in tumor immune surveillance [12, 16]. Previous studies demonstrated that multiple subgroups of CD4^+^ T cells such as Treg cells, Th1 cells, Th17 cells, Th22 cells and Th9 cells play important immune regulatory roles in the pathogenesis of malignant pleural effusion (MPE) [13, 23–28]. Since major subtypes of CD4^+^ T cells contained Th1, Th2, Th17 and Treg cells, the percentages of Th1, Th2, Th17 and Treg cells in malignant ascites were determined in our study. Th1 cells increased significantly in peritoneal fluid compared with peripheral blood. Th1 cells accounted for the highest proportion of CD4^+^ T cells in peritoneal fluid. All these indicated Th1 cells was involved in the development of malignant ascites. Th1 cell was found within the tumor microenvironment. Previous studies showed high numbers of tumor-infiltrating Th1 cells has been identified as a good prognostic marker in many types of cancers [9]. Th1 cells enhance the functions of cytotoxic T lymphocyte (CTL), and recruit natural killer (NK) cells and type I macrophages to tumor microenvironment, which generates antitumor immune surveillance [9]. However, Th1 cells display tumor-promoting roles in some other types of cancers, such as chronic myelogenous leukemia and colorectal carcinoma [9]. The details of Th1 cells in development of malignant ascites have been not reported until now; herein, we aimed to evaluate the effects of Th1 cells on the pathogenesis of malignant ascites. The accumulation of Th1 cells in peritoneal fluid was observed in malignant ascites, and Th1 cells inhibited the growth of tumor cells in vitro. Interferon γ, a hallmark of Th1 lymphocytes [12, 17–19], plays a key role in the development and function of Th1 cells [17, 19]. Our in vitro experiment demonstrated that Th1 cells inhibited the growth of tumor cells by releasing IFN-γ. Then, the mice with the disruption of IFN-γ showed IFN-γ inhibited the formation of malignant ascites and reduced peritoneal permeability. In addition, IFN-γ prolonged survival time in mouse model of malignant ascites. All these demonstrated that IFN-γ secreted by recruited Th1 cells displayed antitumor properties in murine model of malignant ascites. Lin H et al. found that elevated Th1 cell numbers in MPE predominantly produce IFN-γ and IFN-γ promoted the formation of MPE and mouse death in IFN-γ^−/−^ mice [13]. The difference between our results and Lin’s study attributed to different models of serous membrane effusion, different types of tumors and different stages of serous membrane effusion. Obviously, IFN-γ is a crucial cytokine implicated in anti**-**tumor immunity. IFN-γ possesses pro-apoptotic effects on tumor cells, facilitate Th1**-**driven cytotoxic T-cell response, promotes myeloid cell activation and antigen presentation [17, 19]. In addition, IFN-γ exhibited pro-tumorigenic effects under certain circumstances through novel cellular and molecular inflammatory mechanisms [17, 19]. The anti- and pro-tumorigenic functions of IFN-γ seems to be dependent on the contexts of tumor specificity, microenvironmental factors, and signaling intensity [19].

Chemokines play a vital role in recruitment of leukocytes to tumor microenvironments. Th1 cell migrated into peritoneal cavity in response to a chemokine gradient. Firstly, concentration gradients of CXCL-9 and CXCL-10 between ascites and peripheral blood were observed. Further study demonstrated that CXCR3 ablation inhibited the migration of Th1 cell into peritoneal cavity using CXCR3-deficient mice. All these indicated CXCL-9,10/CXCR3 axis was required for Th1 cell trafficking from peripheral blood to peritoneal cavity in malignant ascites. Simultaneously, our study showed that knockdown of CXCR3 expression had no significant effect on the formation of malignant ascites and the growth of tumor in peritoneal cavity in vivo. CXCR3 is expressed on natural killer (NK) cells, B cells, and microvascular endothelial cells [29]. It is well-known that NK cell and B cells are involved in tumorigenesis and tumor progression in many malignancies. CXCR3 plays a crucial role in the chemotaxis of Th1 cells, NK cells and B cells, which leads to complex and divergent effects in the pathogenesis of tumor [30]. In addition, CXCR3 is a double-edged sword in tumor progression [31].

In conclusion, our study illustrated that Th1 cells migrated from peripheral blood to peritoneal cavity through visceral peritoneum, and IFN- γ produced by Th1 cells in peritoneal fluid inhibited the development of malignant ascites. Hence, manipulation of Th1 cells or IFN-γ will provide a therapeutic candidate against malignant ascites.

## Materials and methods

### Detailed materials and methods were provided in the online supplement

#### Animal models of malignant ascites

A rat model of malignant ascites was established through intraperitoneal administration of Walker 256 cell, a rat breast carcinoma cell line (2 × 10^7^ cells per rat) into Sprague-Dawley rats [32, 33]. Murine models of malignant ascites were made through intraperitoneal injection of H22 cell, a murine hepatic carcinoma cell line (1 × 10^6^ cells per mouse) or S180 cells (1 × 10^6^ cells per mouse), a murine sarcoma cancer cell line [34–36]. All animal studies were approved by the institutional animal care and use committee of Tongji Medical College, Huazhong University of Science and Technology.

#### Sample collection and processing

Samples of ascites and serum were obtained from patients with malignant ascites and animal models of malignant ascites. The samples were collected in heparin-treated tubes, and then subjected to further analysis. Samples of enrolled patients were obtained during initial paracentesis of the patients with malignant ascites (17 patients) and the controls (17 patients). In addition, the samples were also collected from animal models of ascites when animal models were successfully created. The samples were incubated with suitable antibodies, and then analyzed on BD LSRFortessa X-20. Data were analyzed using FlowJo 10.5.3.

### The effect of Th1 cells and IFN-γ on the growth of tumor cells in vitro

Naïve CD4^+^ T cells were isolated from a single-cell suspension, which was prepared from a 6-week-old C57/BL6 mouse spleen using the naïve CD4^+^ T Cell Isolation Kit. Cell separation was performed either manually with MACS Columns or automatically with the auto MACS Pro Separator. Then, naïve CD4^+^ T cells were incubated with IL-2, IL-12, and anti-IL-4 to differentiate into Th1 cells. Tumor cells (H22 or S180 cells) were co-cultured with Th1 cells using the trans-well culture system [37]. The effects of Th1 cells on the growth of tumor cells were evaluated by cell proliferation assay and apoptosis assay [38]. In addition, the effects of IFN-γ on the growth of tumor cells were determined by cell proliferation assay and apoptosis assay [38].

### The effects of IFN-γ on the formation of malignant ascites and the growth of tumor cells in vivo

Tumor cells (H22 cells and S180 cells) were injected intraperitoneally into IFN-γ^−/−^ mice or wild-type (WT) mice; then the effects of IFN-γ on the formation of malignant ascites were determined through the weight of peritoneal fluid, peritoneal permeability. The effects of IFN-γ on the growth of tumor cells were evaluated by gross pathology, histology, survival and positron emission tomography and computed tomography (PET-CT) images.

### The effect of CXCR3 on the migration of Th1 cells into peritoneal cavity

Th1-associated chemokines and chemokine receptors were determined in peritoneal fluid and peripheral blood by enzyme-linked immunosorbent assay (ELISA). Tumor cells were injected intraperitoneally into CXCR3^−/−^ mice or wild-type mice to create murine models of malignant ascites; then the formation of malignant ascites and the growth of the tumors in peritoneal cavity were determined. Importantly, the percentage of Th1 cells in CD4^+^ T cells was determined in peripheral blood and peritoneal fluid. The distribution of Th1 cells in parietal peritoneum and visceral peritoneum was determined using immunohistochemical staining [39–41].

## Supplementary information

supplementary

## Data Availability

The datasets supporting the conclusions of this article are included within the article.
