# Supplementary material for: Interferon-γ secreted by recruited Th1 cells in peritoneal cavity inhibits the formation of malignant ascites
Source: Cell Death Discov. 2023 Jan 23;9:25. doi: 10.1038/s41420-023-01312-5 (PMC9870858; doi:10.1038/s41420-023-01312-5)
Supplement: Supplementary file 1 — supplementary [file 41420_2023_1312_MOESM1_ESM.pdf]

Supplementary Information

Interferon-γ secreted by recruited Th1 cells in peritoneal cavity inhibits the formation of malignant ascites

Chang Liu[1*], Zhuanglong Xiao[1*], Li Du[1*], Shenghua Zhu[1], Hongyu Xiang[1], Zehui Wang[1], Fang Liu[2], Yuhu Song[1]

[1]Division of Gastroenterology, Union Hospital, Tongji Medical College, Huazhong University of Science and Technology, Wuhan 430022, China;

[2]Institute of Hematology, Union Hospital, Tongji Medical College, Huazhong University of Science and Technology, Wuhan 430022, China

These authors contributed equally: Chang Liu, Zhuanglong Xiao, Li Du

Corresponding author:

Dr. Yuhu Song

Division of Gastroenterology, Union Hospital, Tongji Medical College, Huazhong University of Science and Technology, Wuhan 430022, China; Email: yuhusong@163.com; Telephone: 0086-27-85726678; Fax: 0086-27-85726057.

Keywords: Th1; malignant ascites**;** IFN-γ; CXCR3

Word count: 4675

Number of figures and tables: 6 figures, 0 table

Conflict of Interest: The authors declare that they have no conflict of interest.

Financial support statement: This work was supported by National Natural Science Foundation of China (No.82070631) and Science and Technology Department, Hubei Provincial People's Government (No.2020CFB421).

Materials and methods

Reagents

See Supplementary Methods for detailed experimental methods and reagents used.

Cells

Walker 256 cancer cell (a rat breast carcinoma cell line)(1), H22 cell (a murine hepatic carcinoma cells)(2), or S180 cells (a murine Sarcoma cancer cell line)(3) were purchased from American Type Culture Collection (ATCC). These cell lines were maintained in RPMI 1640 medium supplemented with 10% fetal bovine serum (FBS), penicillin, and streptomycin in a humidified atmosphere containing 5% CO2 at 37℃.

Animals

C57BL/6 mice and Sprague-Dawley rats were purchased from Beijing Vital River Laboratory Animal Technology Co. Ltd. (Beijing, China). Interferon-gamma (IFN-γ) gene-knockout mice (J002287) were kindly provided by Professor Minjun Ji (Nanjing Medical University, Nanjing, China)(4, 5). CXCR3 knockout mice were generated by Nanjing GemPharmatech Company using CRISPR/Cas9-mediated gene engineering. $CXCR3^{-/-}$ mice were created by the deletion of Exon 2. All animals were housed in specific pathogen-free (SPF) animal facility. The protocol of animal treatment used in this study was approved by the institutional animal care and use committee of Tongji Medical College, Huazhong University of Science and Technology (Wuhan, China).

Humans

In this study, peritoneal fluid was obtained during diagnostic paracentesis in patients with malignant ascites at Union Hospital of Huazhong University of Science and

Technology (Wuhan, China) between November 2017 and October 2021. The following inclusion criteria were used: (a) patients with malignant ascites; (b) patients who had received diagnostic paracentesis; and (c) patients who consented to participate. The exclusion criteria were the following: (a) patients who have received anti-cancer therapy in the last three months; (b) patients with autoimmune diseases, severe immune deficiencies; (c) patients with long term (current) use of antibiotics; (d) patients with the infection in ascitic fluid. The study was conducted according to the principles of the Declaration of Helsinki, and the protocol was approved by the institutional ethics board review and registered at www.chictr.org.cn (ChiCTR-BOC-17011724).

Animal models of malignant ascites

Animal models (rats or mice) of malignant ascites were established by intraperitoneal injection of tumor cells. For murine models of malignant ascites, the mice were anaesthetized and received intraperitoneal injections of 100 ml PBS containing 1×10^6 H22 cells(2, 6) or S180 cells(7). Experimental mice and littermate controls were sex-matched, weight-matched (20-25 g), and age-matched (6-12 weeks). The formation of ascites occurred approximately 2 wk following intrapleural injections of tumor cells. Peritoneal fluid, peritoneum, peritoneal tumors were analyzed in animal models of malignant ascites. The observation of survival advantage included 10 mice per group of wild-type and IFN-$\gamma^{-/-}$ mice. To determine the survival of the animals, the state of mice was observed and the time of death was recorded. Mice without a heartbeat and

no breathing were determined to be dead. For rat model of malignant ascites, 2×10^7 Walker256 cells were administrated into intraperitoneal cavity (8, 9).

Sample Collection and Processing

In patients with malignant ascites and the control, 4 ml of peripheral blood was collected and more than 100 ml of peritoneal fluid were obtained during paracentesis. In animal models of malignant ascites, the animals were sacrificed when the models were successfully established. Blood was drawn from the retro-orbital veins, peritoneal fluid was gently aspirated using a pipette/a 3-ml syringe. The specimens of ascites and peripheral blood were collected in heparin-treated tubes, centrifuged at 3000 rpm for 10 min. After the supernatant was discarded, cell pellets of ascites and blood were resuspended in PBS. Mononuclear cells were isolated by Ficoll paque plus (GE Life Science) to determine T cell subsets.

Flow Cytometry

For intracellular detection of cytokines, cells were stimulated for 4-6 h at 37 °C with Cell stimulation Cocktail, and then surface-stained with the indicated antibodies (Supplementary Methods) in PBS for 30 min at 4°C. Cells were resuspended in a fixation/permeabilization solution. Then, the cells were washed with permeabilization buffer and incubated with anti-IFN-γ mAbs for 30 min at 4°C. Finally, the cells were resuspended with PBS for flow cytometric analysis. Data were examined using FlowJo 10.5.3.

Elisa

The concentrations of CCL3, CCL4, CCL5, CXCL9, CXCL10, CXCL11, VEGF and ANG-2 in peritoneal fluid and peripheral blood were determined using commercially available Elisa kits. ELISA was performed according to the manufacturer's instructions. The ELISA kits were shown in Supplementary Table 1.

Immunohistochemistry Staining

Peritoneal tissues were fixed in 4% paraformaldehyde, embedded with paraffin. The paraffin-embedded tissues were cut into 5-μm thick sections that were mounted on glass slides. The sections were incubated with primary antibodies as recommended by the manufacture. After washing, the slides were incubated with suitable secondary antibodies. Staining process was performed using Two step immunohistochemistry test kit (BOSTER Biological Technology) according to the manufacturer's instructions.

Vascular Permeability Assay

Peritoneal vascular permeability in mice with malignant ascites was determined by Evans blue dye method (10, 11). The mice were injected intravenously with 100 μL 6.25 mg/mL Evans blue dye (Sigma, St Louis) via the tail vein when murine models of malignant ascites were successfully made. 30 minutes later, ascitic fluid and peripheral blood were collected. Then, Evans blue levels in peritoneal fluid and peripheral blood were determined by measuring absorbance at 630 nm in a spectrophotometer.

Positron emission tomography (PET) scanning

The mice were fasted for 12h prior to image acquisition. Approximately 200 ± 10 μCi 18-fluoro-6-deoxy-glucose (FDG) was injected intravenously into mice. After 60 min of FDG uptake, mice were anesthetized with 2% isoflurane and placed on scanning bed. PET/CT images were obtained with the static mode for 10min followed by CT scan of normal mode by the Trans PET Discoverist 180 system (Raycan Technology Co., Ltd, Suzhou, China). The PET images were reconstructed using the three-dimensional (3D) OSEM method with a voxel size of 0.5×0.5×0.5 mm^3. CT images were reconstructed using FDK algorithm with 256×256×256 matrix. Images were displayed with software Carimas (Turku PET Center, Turku, Finland). The total lesion glycolysis (TLG) was defined as the product of the metabolic tumor volume and the median standardized uptake value.

Naive $CD4^+$ T cell isolation and In vitro differentiation

Naive $CD4^+$T cells were extracted from mouse spleen using fluorescence activated cell sorting. Firstly, 24-well plates were coated with 4 μg/ml anti-CD3 and 2μg/ml anti-CD28 before T cell isolation. Secondly, the C57BL/6 mice were anesthetized; their spleens were harvested and cut into small pieces, then were grinded mechanically and filtered. Mononuclear cells were isolated from the filtrate by using mononuclear cell isolation solution. Thirdly, naive $CD4^+$ T cells were sorted out using mouse naive $CD4^+$ T cell isolation kit (Miltenyi). After counting, the cells were added into the 24 well plate with CD3 and CD28, then IL-2 (10 ng/ l), IL-12 (5 ng/ml) and anti-IL-4 (10 μg/ml) were added to stimulate naive $CD4^+$ T cells to differentiate into Th1 cells. After three days, the medium was changed, and IL-2 (10 ng/ml) was added

to the medium. Five days later, flow cytometry was performed to determine the expression of CD4 and IFN-γ to identify the purity of the Th1 cells.

The effect of Th1 cells or IFN-γ on the growth of tumor cells *in vitro*

To determined the effect of Th1 cells on the growth of tumor cells *in vitro*, naive $CD4^+$ T cells/Th1 cells were co-cultured with tumor cells in Transwell chamber. Naive $CD4^+$ T cells/Th1 cells were grown in the lower chamber with 10^5 cells/well, and $2.5*10^4$ tumor cells were seeded in the upper chamber. The apoptosis and proliferative activity of tumor cells were evaluated after 48 hour-incubation using Apoptosis Detection Kit (AntGene) and Cell Counting Kit-8 (DOJINDO Laboratorise). To investigate the effect of IFN-γ on the growth of tumor cells in vitro, tumor cells were treated with IFN-γ (100 ng/ml) contained into the culture medium. 48 hours later, proliferative activity and the apoptosis were determined. All the operations were performed according to the manufacturer's instructions.

Statistical Analysis

Continuous variables were expressed as mean and standard deviation for normal distribution data and median ± interquartile range if the distribution was skewed. Categorical variables were presented as count (percentage). Differences in means were examined by 2-tailed Student’s t test, or 1-way or 2-way ANOVA with Bonferroni post-tests, as appropriate, and in medians by Mann-Whitney U test or Kruskal-Wallis test with Dunn’s post hoc tests. The correlation of continuous variables by Pearson correlation test. $P < 0.05$ was considered significant. Analyses and plots were done on Prism v5.0 (GraphPad Software).

Key resource table

Reagents or resources	Source	Application	Catalogue number
Antibodies			
Anti -CD4 Rabbit pAb	CST	Immunohistochemistry	25229T
IFN-γ antibody	R&D systems	Immunohistochemistry	AF-585
Rat CXCR3 Alexa Fluor 647-conjugated Antibody	R&D systems	Flow cytometry	FAB8109RV
Human/Mouse/Rat CCR5 APC-conjugated Antibody	R&D systems	Flow cytometry	FAB1802A
Rat CD4 FITC	BD Pharmingen	Flow cytometry	561833
PE Mouse Anti-Rat IFN-gamma	BD Pharmingen	Flow cytometry	559499
Anti-Mouse CD4 FITC	eBioscience	Flow cytometry	11-0042-82
Anti-Mouse IFN-gamma APC	eBioscience	Flow cytometry	17-7311-81
Hu/NHP IFN-Gama APC	BD Pharmingen	Flow cytometry	551385
PE-Mouse Anti-Human IL-17A	BD Pharmingen	Flow cytometry	580486
FITC-Mouse Anti Human CD4	BD Pharmingen	Flow cytometry	555346
APC-Mouse Anti Human IFN-gamma	BD Pharmingen	Flow cytometry	554702
APC-Cy7-Mouse Anti Hu CCR5	BD Pharmingen	Flow cytometry	557755
PE Mouse Anti-Human CXCR3	BD Pharmingen	Flow cytometry	557185
PE-Cy 7 Mouse anti human CD25	BD Pharmingen	Flow cytometry	557741
Alexa Fluor 647 Mouse anti Human CD127	BD Pharmingen	Flow cytometry	558598

PE-Cy 7 Mouse anti human IL-4	BD Pharmingen	Flow cytometry	560672
Cell stimulation Cocktail	eBioscience	Flow cytometry	00-4975-93
MS CD62L PE	BD Pharmingen	Flow cytometry	561918
Cytofix/CytopermSoln Kit	BD Pharmingen	Flow cytometry	554714
Purified NA/LE Rat Anti-Mouse IL-4	BD Pharmingen	Magnetic Activated Cell Sorting	554385
**Elisa kit**			
Human CCL3 (MIP-1a) ELISA Kit	MultiSciences	Elisa	EK161-24
Human CCL4 (MIP-1 beta) ELISA Kit	MultiSciences	Elisa	EK162-96
Human CCL5/RANTES ELISA Kit	MultiSciences	Elisa	EK1129-96
Human CXCL9/MIG ELISA Kit	MultiSciences	Elisa	EK1143-96
Human CXCL-10/IP-10 ELISA Kit	MultiSciences	Elisa	EK168-96
Human CXCL11/I-TAC ELISA Kit	MultiSciences	Elisa	EK12071
Human VEGF ELISA Kit	MultiSciences	Elisa	EK183-96
Human Angiopoietin-2/ANG-2 ELISA Kit	MultiSciences	Elisa	EK12151
Mouse ANG-2 ELISA Kit	MeiBiao Biology	Elisa	MB-2855B
Mouse VEGF ELISA Kit	MultiSciences	Elisa	EK-283/2-48
Rat CCL3/MIP-1a ELISA KIT	MultiSciences	Elisa	EK-361-48
Rat CCL5/RANTES ELISA Kit	MultiSciences	Elisa	EK-3129-48
Rat CCL4/MIP-1b ELISA KIT	MB Biology	Elisa	MB-7093B

Rat CXCL9/MIG ELISA Kit	MB Biology	Elisa	MB-7103B
Rat CXCL-10/IP-10 ELISA Kit	MB Biology	Elisa	MB-7100B
Rat CXCL11/I-TAC ELISA Kit	MB Biology	Elisa	MB-7097B
**Chemicals, Peptides, and Recombinant Proteins**			
Cell Counting Kit-8	DOJINDO Laboratorise	Co-culture	CK04
Apoptosis Detection Kit	AntGene	Co-culture	ANT002
LS Separation columns	MiltenyiBiotec	Magnetic Activated Cell Sorting	130-042-401
AutoMACS Running Buffer	MiltenyiBiotec	Magnetic Activated Cell Sorting	130-091-221-1
FICOLL PAQUE PLUS	GE Life Sciences	Magnetic Activated Cell Sorting	17144002
Naive CD4+ T Cell Isolation Kit, mouse	MiltenyiBiotec	Magnetic Activated Cell Sorting	130-104-453
Mouse CD28	BD Pharmingen	Magnetic Activated Cell Sorting	553294
Ms CD3e	BD Pharmingen	Magnetic Activated Cell Sorting	553057
Recombinant Mouse IL-2 Protein	R&D systems	Magnetic Activated Cell Sorting	402-ML-020
Recombinant Mouse IL-12 Protein	R&D systems	Magnetic Activated Cell Sorting	419-ML-010
Evans blue	Sigma	Membrane Permeability	E-2129
Two step immunohistochemistry test kit	BOSTER BIOLOGICAL TECHNOLOGY	Immunohistochemistry	SV0002
Hematoxylin-Eosin Staining Kit	BOSTER BIOLOGICAL	Immunohistochemistry	AR1180

	TECHNOLOGY		
Carbon tetrachloride	Makclin	Rat model	C822982
Olive oil	Makclin	Solvent for CCl_4	O815211
Sirius red staining	Solarbio	ECM depostion	G1470-2
DAPI	Sigma	IF	28718-90-3
Mouse Direct PCR Kit	Bimake	Gene identification	B40015
Recombinant Murine IFN-γ	PeproTech	Cell experiment	315-05
RPMI-1640 basic	Gibco	Cell culture	C11875500BT
Fetal Bovine Serum	Gmini	Cell culture	900-108
Penicillin- Streptomycin	Sigma	Cell culture	V900929
**Software and Algorithms**			
Adobe Photoshop CS6	Adobe	Photo	Version19.1.2
NIS-Elements Viewer	Laboratory Imaging	IF	Version3.20.02
ImageJ	National Institutes of Health	IHC	Version1.51j8
GraphPad Prism	GraphPad Software	Photo	Version5.0.1
FlowJo	Becton, Dickinson & Company	Flow cytometry	Version10.0.7

Supplementary Figure 1

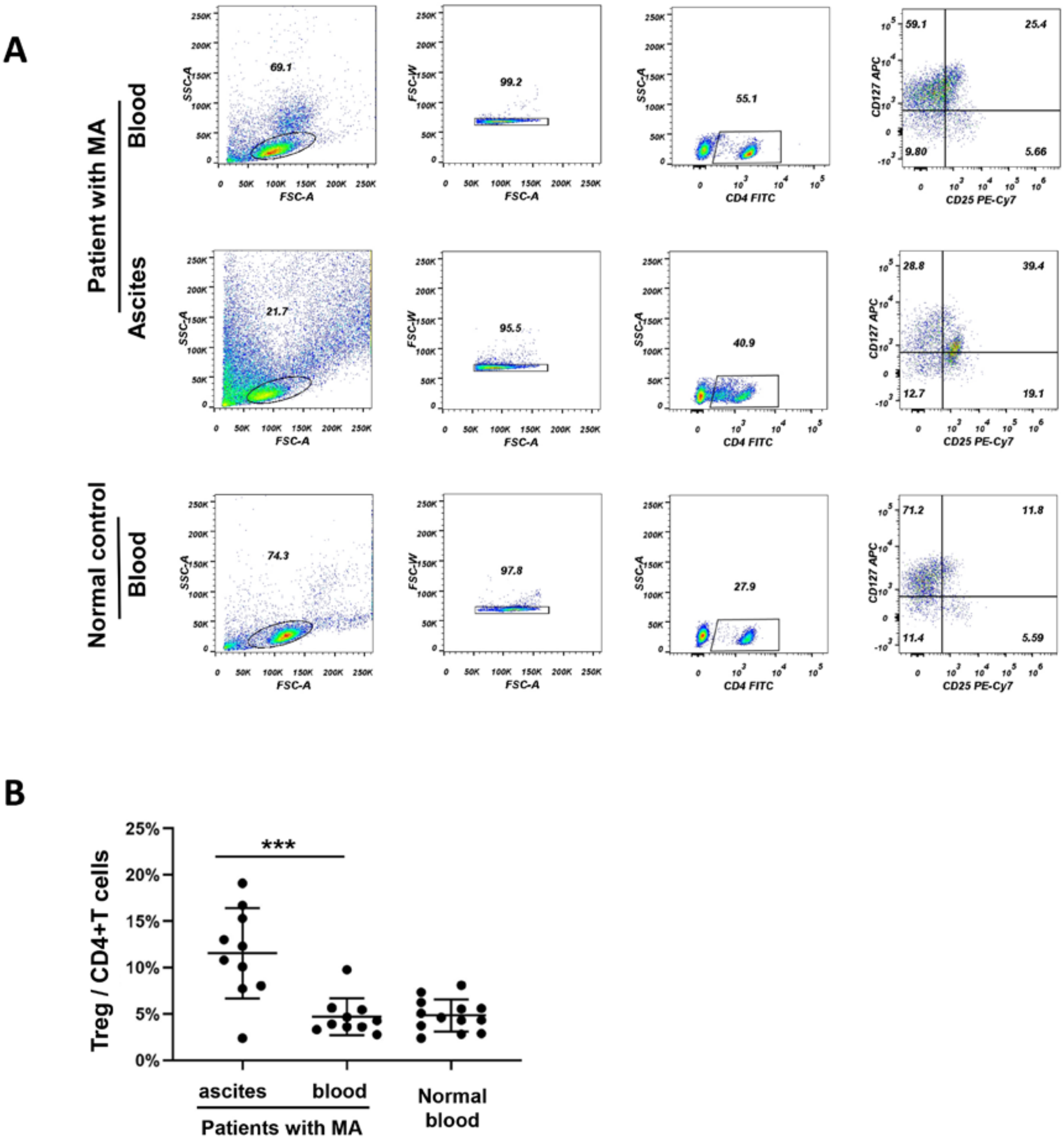

Figure.S1. Treg cells in patients with malignant ascites.

A. Representative dot plots from a patient with malignant ascites and a healthy control showing flow cytometric analysis of Treg ($CD4^+CD25^+CD127^-$) cells;

B. the percentages of Treg cells in peritoneal fluid, peripheral blood in patients with malignant ascites, ***: p≤0.001. MA: malignant ascites.

Supplementary Figure 2

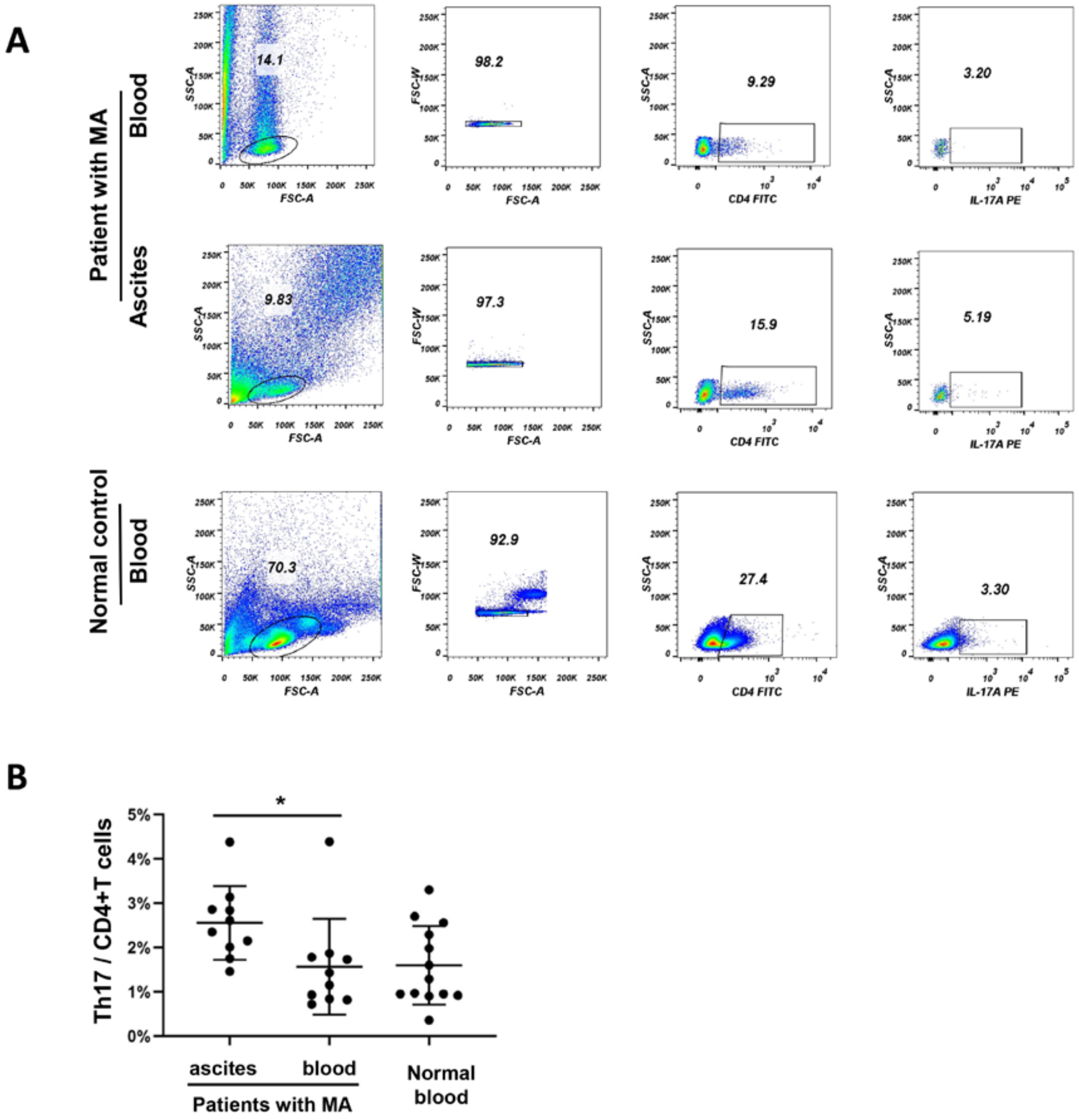

Figure.S2. Th17 cells in patients with malignant ascites**.**

A. Representative dot plots from a patient with malignant ascites and a healthy control showing flow cytometric analysis of Th17 ($CD4^{+}$ $IL\text{-}17^{+}$) cells;

B. the percentages of Th17 cell in peritoneal fluid, peripheral blood in patients with malignant ascites , *: p≤0.05. MA: malignant ascites.

Supplementary Figure 3

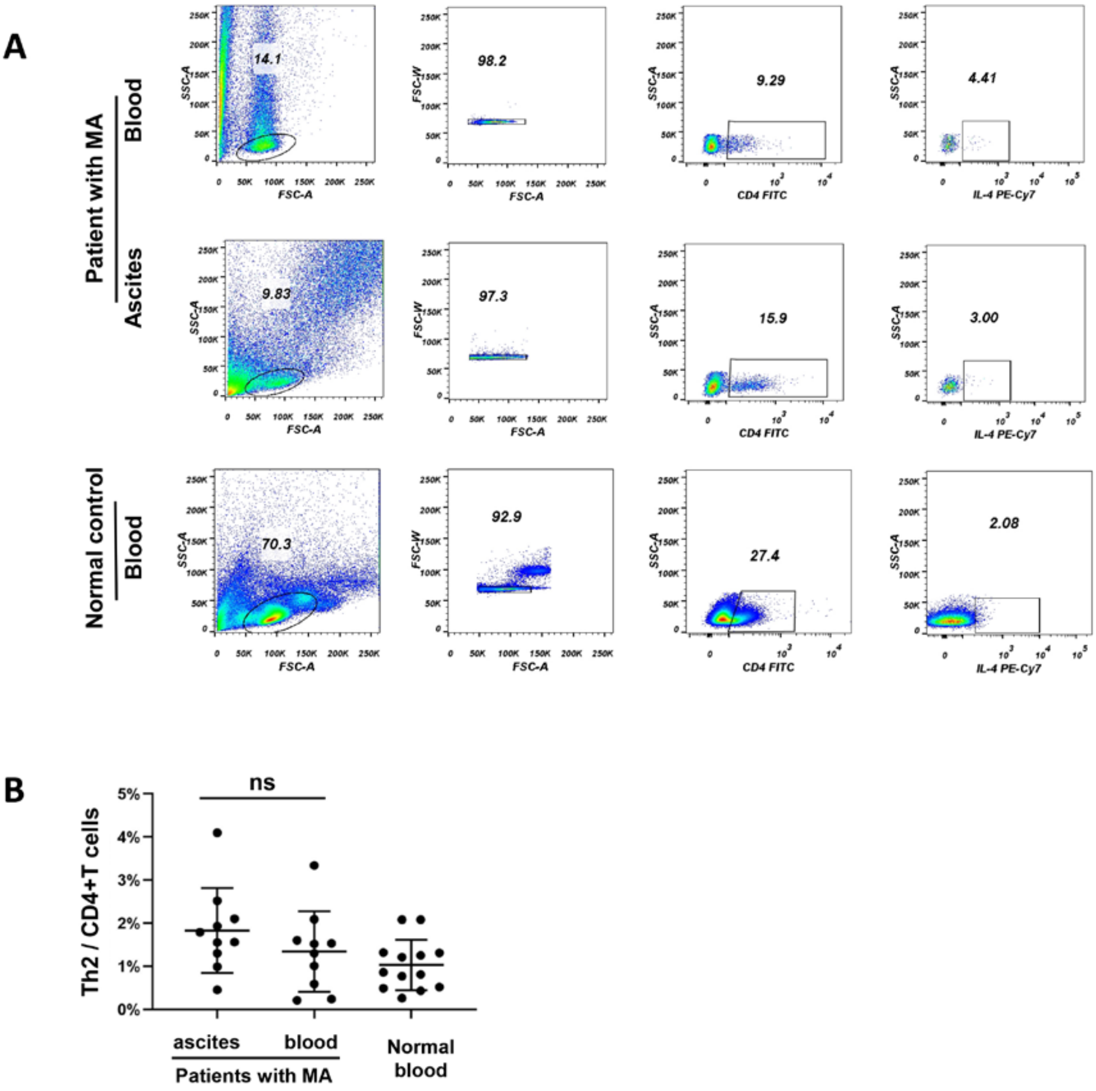

Figure.S3. Th2 cells in patients with malignant ascites**.**

A. Representative dot plots from a patient with malignant ascites and a healthy control showing flow cytometric analysis of Th2 ($CD4^{+}$ IL-4^{+}) cells;

B. the percentages of Th2 cells in peritoneal fluid, peripheral blood in patients with malignant ascites, ns: not significant (P>0.05). MA: malignant ascites.

Supplementary Figure 4

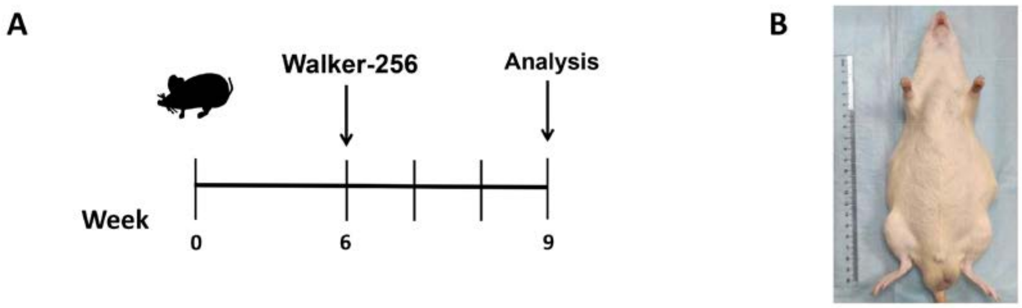

Figure.S4. The establishment of rat model with malignant ascites. Rat model of malignant ascites was established through intraperitoneal injection of a rat breast carcinoma cell, Walker 256 cell.

A. Experiment design for a rat model of malignant ascites

B. representative photograph from a rat with malignant ascites showing marked abdomen expansion at 21 days after the administration of tumor cells.

Supplementary Figure 5

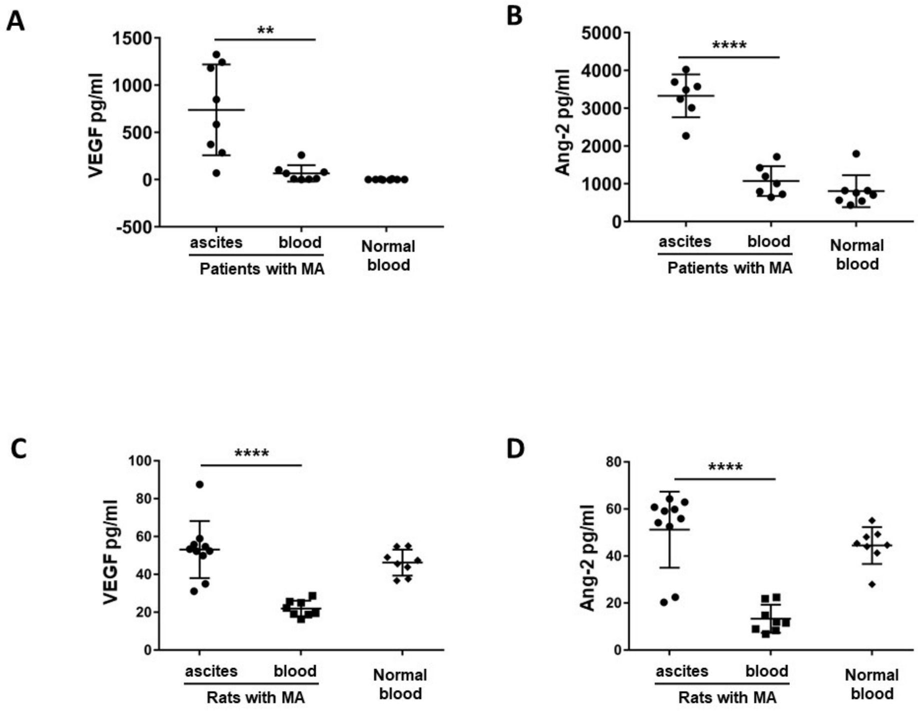

Figure.S5. the concentration of VEGF and ANG-II increased in peritoneal fluid compared with corresponding blood.

A. In patients with malignant ascites, the level of VEGF increased in peritoneal fluid compared with corresponding blood;

B. in patients with malignant ascites, increased Ang-II level was observed in peritoneal fluid compared with corresponding blood;

C. in rat model of malignant ascites, the level of VEGF increased in peritoneal fluid compared with corresponding blood;

D. in rat model of malignant ascites, increased Ang-2 level was observed in peritoneal fluid compared with corresponding blood.

MA: malignant ascites; *: p≤0.05, **: p≤0.01, ***: p≤0.001, ****: p≤0.0001, ns: no significant.

Supplementary Figure 6

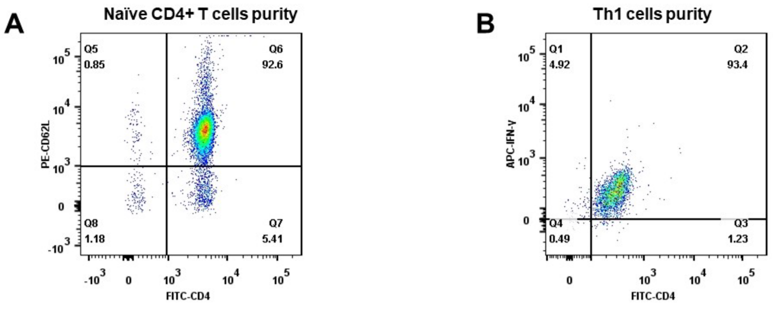

Fig.S6. Naive CD4+ T cells were isolated, and differentiated into Th1 cells. Then, the purity of naive CD4+ T cells and Th1 cells were determined through flow cytometry.

A. Representative dot plots **of** flow cytometric analysis showing the purity of isolated naïve CD4+ T cells (CD4+ CD62L+) was > 90%;

B. Representative dot plots of flow cytometric analysis showing the purity of Th1 cells was > 90%.

Supplementary Figure 7

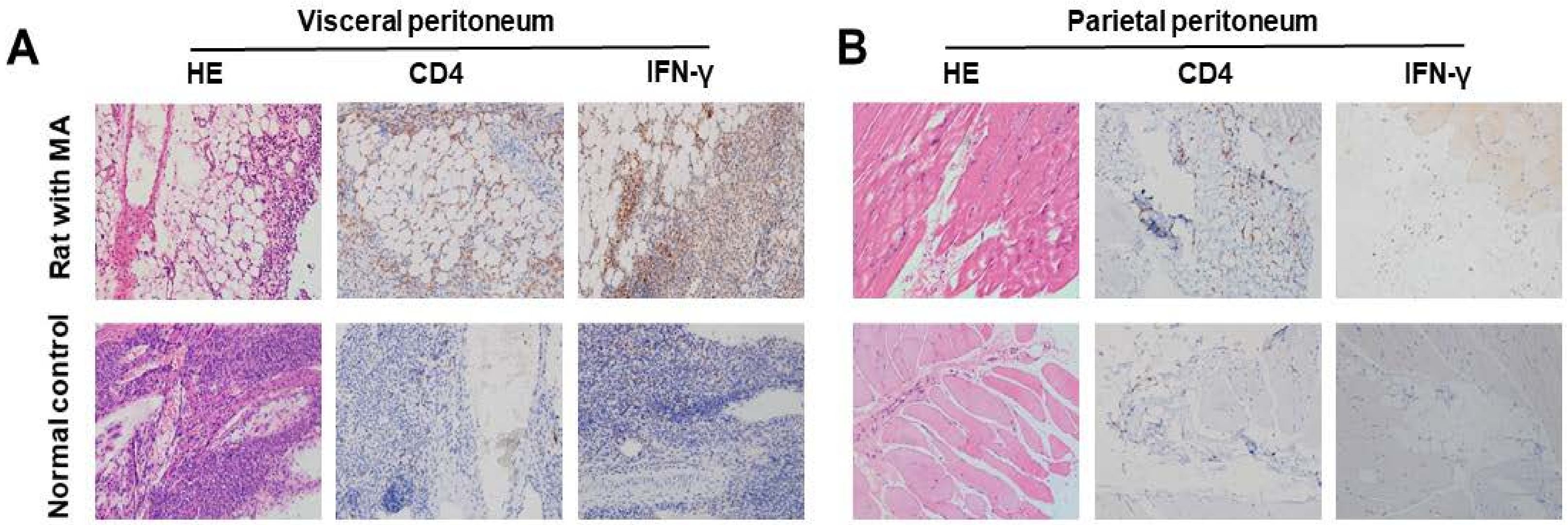

Fig.S7. Th1 cells ($CD4^+IFN\text{-}\gamma^+$) in visceral and parietal peritoneum in rat model of malignant ascites (MA)

A. Immunohistochemical staining showed most of Th1 ($CD4^+IFN\text{-}\gamma^+$) cells were found in visceral peritoneum.

B. immunohistochemical staining of CD4 and IFN-γ showed few of Th1 ($CD4^+IFN\text{-}\gamma^+$) cells were found in parietal peritoneum.

Supplementary Figure 8

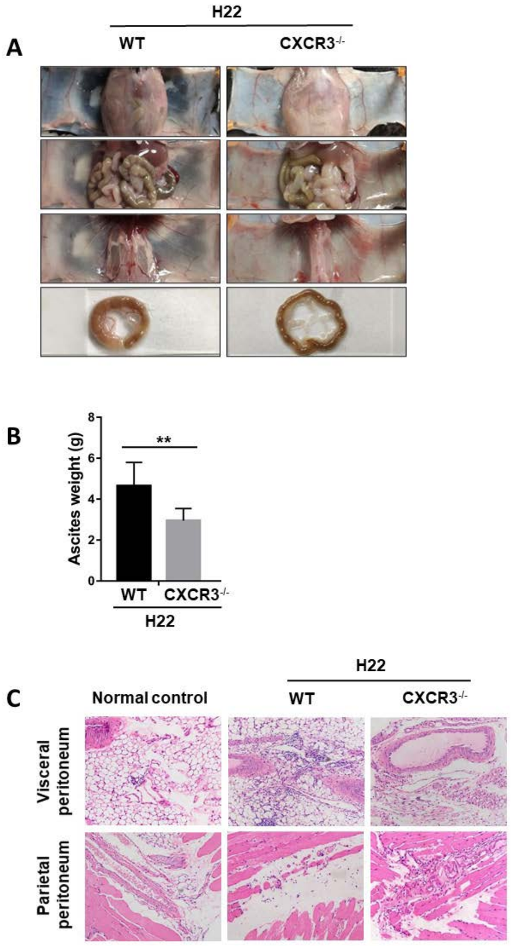

Fig.S8. The effect of CXCR3 deficiency on the growth of peritoneal carcinomatosis and the formation of malignant ascites in H22 cell-induced murine model of malignant ascites.

A. Marked abdomen expansion and multiple tumor foci were observed in WT and $CXCR3^{-/-}$ mice at 14 days after intraperitoneal injection of H22 cells. Most of tumor foci were located in visceral peritoneum.

B. ascites weight was not increased in $CXCR3^{-/-}$ mice compared with wild-type controls, **: $p \leqslant 0.01$;

C. Representative HE images of parietal and visceral peritoneum from $CXCR3^{-/-}$ mice and wild-type mice injected with H22 cell intraperitoneally.

Supplementary Figure 9

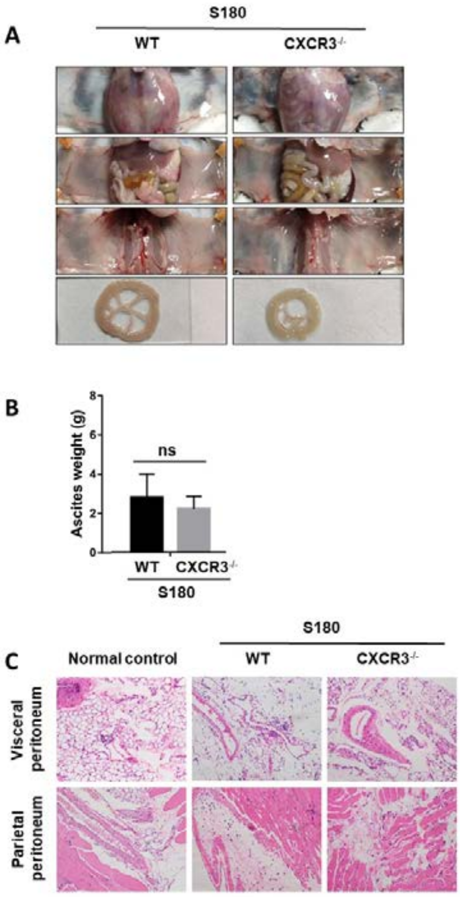

Fig.S9. The effect of CXCR3 deficiency on the growth of peritoneal carcinomatosis and the formation of malignant ascites in S180 cell-induced model of malignant ascites.

A. Marked abdomen expansion and multiple tumor foci were observed in WT and $CXCR3^{-/-}$ mice at 14 days after intraperitoneal injection of S180 cells. Most of tumor foci was located in visceral peritoneum;

B. no significant difference in ascites weight between WT mice and $CXCR3^{-/-}$ mice, ns: not significant;

C. Representative HE images of parietal and visceral peritoneum from $CXCR3^{-/-}$ mice and wild-type mice injected with S180 cell intraperitoneally.

References

1. Muta M, Matsumoto G, Nakashima E, Toi M. Mechanical analysis of tumor growth regression by the cyclooxygenase-2 inhibitor, DFU, in a Walker256 rat tumor model: importance of monocyte chemoattractant protein-1 modulation. Clin Cancer Res 2006;12:264-272.
2. Yu J, Ji HY, Liu C, Liu AJ. The structural characteristics of an acid-soluble polysaccharide from Grifola frondosa and its antitumor effects on H22-bearing mice. Int J Biol Macromol 2020.
3. Deng Z, Gao S, Xiao X, Yin N, Ma S, Li W, Li Y. The effect of earthworm extract on mice S180 tumor growth and apoptosis. Biomed Pharmacother 2019;115:108979.
4. Dalton DK, Pitts-Meek S, Keshav S, Figari IS, Bradley A, Stewart TA. Multiple defects of immune cell function in mice with disrupted interferon-gamma genes. Science 1993;259:1739-1742.
5. Du X, Wu J, Zhang M, Gao Y, Zhang D, Hou M, Ji M, et al. Upregulated expression of cytotoxicity-related genes in IFN-gamma knockout mice with Schistosoma japonicum infection. J Biomed Biotechnol 2011;2011:864945.
6. Zhang J, Wang X, Lu H. Amifostine increases cure rate of cisplatin on ascites hepatoma 22 via selectively protecting renal thioredoxin reductase. Cancer Lett 2008;260:127-136.
7. Deng Z, Gao S, Xiao X, Yin N, Li YJB, Pharmacotherapy. The effect of earthworm extract on mice S180 tumor growth and apoptosis. 2019;115:108979.
8. Badraoui R, Rebai T. Effect of malignant ascites on antioxidative potency of two tumoral cells-induced bone metastases: Walker 256/B and MatLyLu. Exp Toxicol Pathol 2012;64:65-68.
9. Zhang Y, Lou JW, Zhang Q, Li ZL, Bao BH, Cao YD, Yao WF, et al. Determination of kansuiphorin C and kansuinin A in rat feces using UFLC-MS/MS and its application in the comparative excretion study on normal and malignant ascites rats. J Pharm Biomed Anal 2019;170:254-263.
10. Lin H, Tong ZH, Xu QQ, Wu XZ, Wang XJ, Jin XG, Ma WL, et al. Interplay of Th1 and Th17 cells in murine models of malignant pleural effusion. Am J Respir Crit Care Med 2014;189:697-706.
11. Lo CW, Chen MW, Hsiao M, Wang S, Chen CA, Hsiao SM, Chang JS, et al. IL-6 trans-signaling in formation and progression of malignant ascites in ovarian cancer. Cancer Res 2011;71:424-434.
